# Perioperative antibiotic prophylaxis in spinal surgery

**DOI:** 10.1051/sicotj/2021029

**Published:** 2021-05-10

**Authors:** Ahmed Shawky Abdelgawaad, Mohammed Hassaan Mohamed El Sadik, Khalid Mohammed Hassan, Mohammad El-Sharkawi

**Affiliations:** 1 Department of Orthopaedic and Trauma Surgery, Assiut University Hospitals 71515 Assiut Egypt; 2 Spine Center, Helios Klinikum Erfurt Nordhaeuser Street 7 99089 Erfurt Germany

**Keywords:** Antibiotic prophylaxis, Lumbar spinal surgeries, Surgical site infection, First-generation cephalosporin, Clinical audit

## Abstract

*Study design*: Complete audit cycle. *Introduction*: To highlight the unjustified overuse of perioperative antibiotics in clean non-instrumented lumbar spinal surgeries. To convince orthopedic surgeons in a methodological way of local field comparison between common practice on the use of perioperative antibiotics prophylaxis (PAP) in clean non-instrumented lumbar spinal surgeries and the ideal practice according to “The guidelines published by North American Spine Society (NASS)”. *Methods*: A complete audit cycle had been done. One hundred and eight patients underwent clean non-instrumented lumbar spinal surgeries in a tertiary spine center, during the period from the 1st of April to the 31st of June 2017 (primary audit period) and during the period from the 8th of May to the 21st of November 2018 (re-audit period). Group I: audit group (*n* = 54) was given the usual regimen (IV first-generation cephalosporin for 1–6 days, followed by oral antibiotics, till the removal of stitches) and Group II: re-audit group (*n* = 54) received only the IV antibiotics for one day). The study protocol was approved by our institution’s Ethical Committee (17100582). *Results*: This study showed a wide gap between international standards and local prescribing practices and calls for multiple interventions to improve our practice. Out of the 108 patients, only one case (1.85%) developed surgical site infection (SSI) in the audit group (Group I). The difference in infection rates between the two groups was statistically insignificant. *Conclusion*: A single-day postoperative dose of antibiotics effectively prevents postoperative wound infection following non-instrumented lumbar spinal surgery and is not associated with a higher infection rate.

## Introduction

The use of antibiotics for surgical prophylaxis is a standard of care and an important component of all procedures [1]. Surgical antibiotics prophylaxis (SAP) can be defined as “a brief course of an antimicrobial agent initiated before an operation begins in order to reduce intraoperative microbial contamination to a level that will not overpower host defense and lead to infection” [2].

Surgical site infection (SSI) is classified as follows: Superficial incisional, deep incisional, and organ/space [3]. The rate of infection for lumbar discectomy has been reported to be <1% [4], while the rate of infection for non-instrumented fusions has been reported to range from less than 1 up to 5% [5]. For SAP, the current consensus is that antibiotic administration should be started within 30–60 min before skin incision [6]. The current guidelines also suggest that postoperative antibiotics should be discontinued within 24 h after spine surgery [7]. Furthermore, it has been recently proved that prolonged use of antibiotics is often associated with increasing antimicrobial resistance, toxicity and unnecessary cost, and even higher incidence of post-operative infection [8].

In the absence of strict hospital/country guidelines on antibiotic prescription, the authors have observed inconsistency and common overuse of both intravenous (IV) and oral antibiotics extending up to 14 days postoperatively, often with the use of antibiotics combination, both in the Spine Unit of their tertiary care hospital and among colleagues in private practice all over the country. This unjustified overuse is multifactorial. It may be related to the clinician, the procedure, or the patient. For clinicians, it is difficult to shift from the old unsupported practices to the new evidence-based prescriptions. Also, the non-prescription purchase is unregulated in many countries that make antibiotics available to the public cheaply and in large amounts [9–12]. The rationale behind this was common sense that our patients’ hygiene is less than the international standards at which the standard guidelines had been made. Thus, we probably need more antibiotics to further reducing the incidence of postoperative infection or at least getting up to the standards.

In a Cochrane meta-analysis, persuasive interventions to reduce exposure of patients to antibiotics were as effective as restrictive interventions after six months [9]. This work aimed to perform a professional persuasive intervention in form of clinical audit as a method of antibiotic stewardship in surgical antibiotic prophylaxis. The study aimed at convincing orthopedic surgeons that they had wrong believes in a methodological way of head-to-head local field comparison between common practice on the use of perioperative antibiotics prophylaxis (PAP) in clean non-instrumented lumbar spinal surgeries and the ideal practice according to “The guidelines [7] published by North American Spine Society (NASS)”.

## Material and methods

This prospective complete audit cycle involved all patients who underwent non-instrumented lumbar spinal surgeries in the Spine Unit of the Orthopaedic and Trauma Surgery Department of Assiut University, Egypt. The study protocol was approved by the Assiut Medical School Ethical Committee (#17100582). All study participants signed informed consent. We had excluded patients with known hypersensitivity to antibiotics, concomitant steroid therapy, concomitant systemic infection, and immunocompromised patients and those who refused to participate in this audit. Routine erythrocytic sedimentation rate (ESR) and C-reactive protein (CRP) were done for all patients to exclude hidden infections.

### Step 1 (measuring current practice)

**Figure 1:**
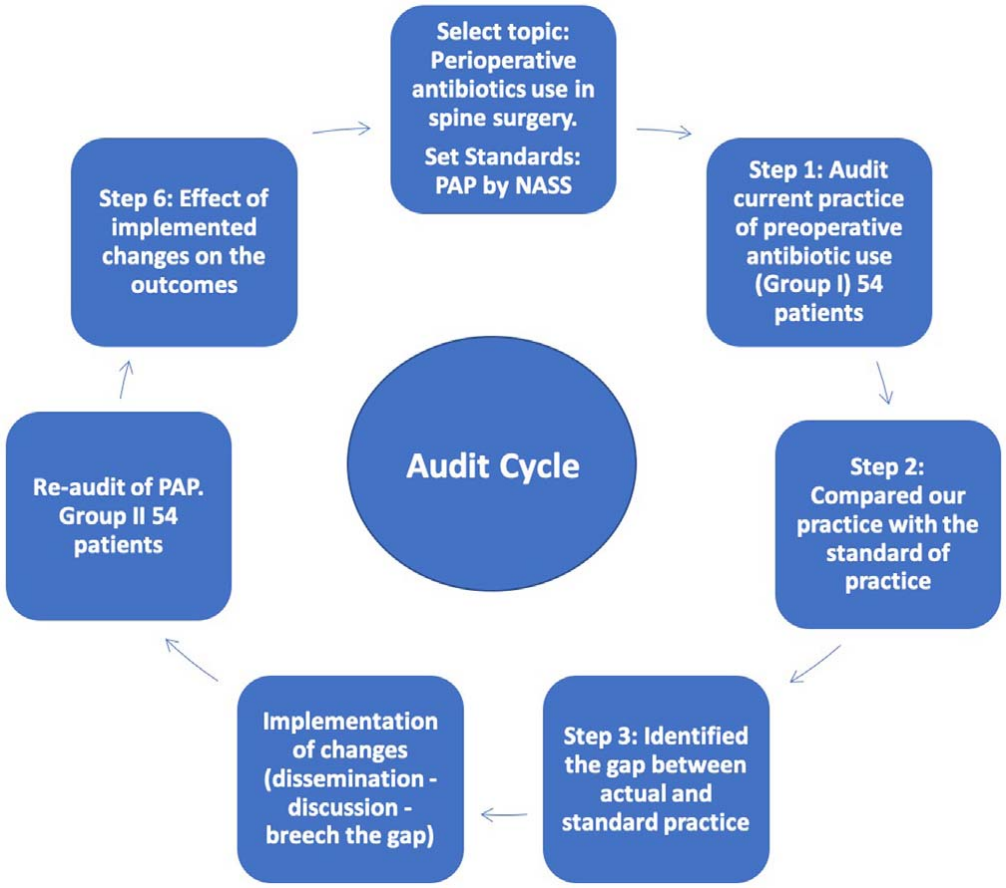
Audit cycle. Step by step discerption of our audit process.

We collect data of 54 patients during the period from the 1st of April to the 31st of June 2017 (Figure 1). In a specially designed audit sheet, we prospectively collect the following data; demographics, intraoperative details, and postoperative antibiotic use. Additionally, the following variables were recorded: gender; age in years; admission, surgery and discharge dates; body mass index; the presence of co-morbidities, and surgical procedures.

Operative data were included; details of the surgical procedure, spinal level, operative time, estimated blood loss (EBL), and intraoperative complications. For patients who received prophylactic antibiotics in the operating room, the following parameters were recorded: type, time (minutes from the incision), and dose of preoperative antibiotics, the aforementioned parameters were also registered for patients who were given antibiotics postoperatively in addition to the duration of its use.

SSI was defined according to the modified CDC (Centers for Disease Control) definitions [13].

### Step 2: We compared our practice with the standard practice NASS (Figure 1)

NASS guideline recommendations were used in this study because it was the most recent available international guideline on the use of PAP in non-instrumented lumbar spinal surgeries at the time of study beginning. Agreements and disparities regarding the type of antibiotics, timing, doses, administration route, and duration were identified (Table 1).

**Table 1:** Disparities between the current practice and the NASS recommendations.

Research question	NASS 2013 recommendations	Current practice
A. Efficacy		
For patients undergoing spine surgery, does antibiotic prophylaxis result in decreased infection rates?	Preoperative prophylactic antibiotics are suggested to decrease infection rates in patients undergoing spine surgery.	Prophylactic antibiotics were given to patients undergoing spine surgeries.
	Grade of recommendation: B	
	For a typical, uncomplicated lumbar laminotomy and discectomy, a single preoperative dose of antibiotics is suggested to decrease the risk of infection and/or discitis.	A single preoperative dose is not the current practice. Prolonged use >48 h was the common practice.
	Grade of recommendation: B	
For patients undergoing spine surgery *without* spinal implants, does antibiotic prophylaxis result in decreased infection rates?	Prophylactic antibiotics are suggested to decrease the rate of spinal infections following un-instrumented lumbar spinal surgery.	Agree
	Grade of recommendation: B	
B. Protocol		
For patients receiving antibiotic prophylaxis prior to spine surgery *without* spinal implants, what are the recommended drugs, their dosages, and time of administration resulting in decreased postoperative infection rates?	Preoperative antibiotic prophylaxis is suggested to decrease infection rates in patients undergoing spine surgery without spinal implants. In these typical, uncomplicated spinal procedures, the superiority of one agent, dose, or route of administration over any other has not been demonstrated. When determining the appropriate drug choice, the patient’s risk factors, allergies, length and complexity of the procedure, and issues of antibiotic resistance should be considered.	Tendency to use third-generation cephalosporins or combination of antibiotics.
	Grade of recommendation: B	
C. Redosing		
For patients receiving antibiotic prophylaxis prior to spine surgery, what are the intraoperative redosing recommendations for the recommended drugs (including dosages and time of administration) resulting in decreased postoperative infection rates?	*Consensus statement*: Intraoperative redosing within 3–4 h may be considered to maintain therapeutic antibiotic levels throughout the procedure. The superiority of one drug has not been demonstrated in the literature. When determining the appropriate drug choice, the patient’s risk factors, allergies, length and complexity of the procedure, and issues of antibiotic resistance should be considered.	Agree
D. Discontinuation		
For patients receiving antibiotic prophylaxis prior to spine surgery, does discontinuation of prophylaxis at 24 h result in decreased, or increased postoperative infection rates as compared to longer periods of administration?	For typical, uncomplicated cases, a single dose of preoperative prophylactic antibiotics with intraoperative redosing as needed is suggested to decrease the risk of SSI.	Prolonged postoperative regimens up to 14 days is the common practice.
	Grade of recommendation: B	
	Prolonged postoperative regimens may be considered in complex situations (i.e., trauma, cord injury, neuromuscular disease, diabetes, or other co-morbidities).	
	Grade of recommendation: C	
E. Body habitus		
For patients receiving antibiotic prophylaxis prior to spine surgery, should the recommended protocol differ based upon body habitus (e.g., body mass index)?	Obese patients are at higher risk for postoperative infection when given a standardized dose of antibiotic prophylaxis.	Prolonged regimen in obese patients is the common practice.
	Despite this conclusion, there is insufficient evidence to make a recommendation for or against recommending a different protocol for patients based upon body habitus.	
	Grade of recommendation: I (insufficient evidence)	

### Step 3: Identify the gap between the actual and standard practice (Figure 1)

And set recommendations to fill this gap. Gaps could be identified regarding the prolonged use of intravenous and oral administration, use of third and fourth-generation antibiotics, and the use of antibiotic combinations. The main disparity was the absence of an institutional standard for perioperative antibiotic prophylaxis in spinal surgery.

### Step 4: Implementation of changes (Figure 1)

Through several discussion sessions with the Head of Spine Unit, staff members, fellows, and residents as well as the Head of the Orthopaedic and Trauma Surgery Department, all the disparities between the common local practice and the international guidelines were presented and discussed in detail. The agreement was made on changing the local SAP protocol and strict adherence by all staff members and spine fellows to the newly implemented local SAP protocol. We also disseminate the new protocol to all our staff, anesthetic team, and residents by a pamphlet for the updated guidelines in addition to a Poster that fitted at the entrance of the operation theater.

This protocol included intravenous administration of a first-generation cephalosporin 30–60 min before surgical incision. In longer procedures, another dose was given after 3 h. Antibiotic was discontinued 24 h postoperatively.

### Step 5: Re-audit (Figure 1)

After about a year later (8th May to the 21st of November 2018) we used the same data collection sheet to collect another 54 patients: All the above data were prospectively recorded on the data collection form. Close observation and monitoring of all operations by one of the researchers (MH) to ensure strict adherence to the SAP protocol. He also monitors antibiotic administration in the postoperative ward and with the patients after discharge from the hospital. Three follow-up visits were scheduled: After two weeks (early postoperative) for suture removal and check of the surgical wound, after one month and after three months (late postoperative) for checking of the surgical wound and searching for any evidence of SSI.

The effect of implemented changes on the outcomes was then evaluated. We compared the preoperative, intraoperative, and postoperative data of the patients in pre-and post-implementation of SAP protocol. Our primary outcome was the incidence of postoperative infections (Figure 1).

### Demographics of the audit and re-audit groups

A total of 108 patients were included in this audit. The demographic characteristics of the patients are shown in (Table 2). The mean age of our patients was 43.2 (23–80) and 43.76 (20–70) years in Groups I (audit) and II (re-audit); respectively. No statistically significant differences were found among the two groups concerning gender, age, smoking habits, and comorbidities, but the body mass index (BMI) was significantly higher in the audit group (*P* = 0.001). Of these 108 surgeries, open discectomy, decompression, and (decompression and discectomy) were performed in 77 (71.3%), 17 (15.7%), and 14 (13%) cases respectively. The average postoperative hospital stay was 3.33 (1–8) and 2.04 (2–3) days in Groups I and II; respectively (Figure 1).

**Table 2:** Characteristics of study participants in the audit and re-audit period.

	Group I		Group II		*P*-value
	Audit group (*N* = 54)		Re-audit group (*N* = 54)		
	*N*	%	*N*	%	
Gender					
Male	37	68.5	42	77.8	0.278
Female	17	31.5	12	22.2	
Age: (years)					
Mean ± SD	43.20 ± 12.13		43.76 ± 11.94		0.811
Range	23.0–80.0		20.0–70.0		
Occupation					
Employee	23	42.6	24	44.4	0.846
Unemployed	31	57.4	30	55.6	
Smoking					
Smoker	24	44.4	19	35.2	0.326
Non-smoker	30	55.6	35	64.8	
BMI					
Mean ± SD	24.45 ± 3.37		22.55 ± 2.19		0.001*
Range	19.0–32.4		19.0–28.0		
Surgery					
Discectomy (*n* = 77)	39		38		0.965
Decompression (*n* = 17)	8		9		
Decompression + discectomy (*n* = 14)	7		7		

Group I: audit group, Group II: re-audit group.

* Statistically significant *p* value.

All patients during the audit period were given 1 gm parenteral antibiotic prophylaxis starting 30 min before surgery, using first-generation cephalosporin. This is in complete accordance with the current guidelines [14]. However, there was obvious overuse of postoperative prophylactic antibiotics in the form of extended duration, the use of the antibiotic combination, as well as the frequent use of second-generation cephalosporins. The duration of IV antibiotics was extended beyond 24 h postoperatively up to six days in some cases. This was followed by oral antibiotics up to two weeks postoperatively in all patients (until removal of sutures). The type of antibiotic was also not strictly defined nor consistent; so, variation between first, second-generation cephalosporins and Amoxicillin–clavulanic acid was used.

## Results

**Figure 2:**
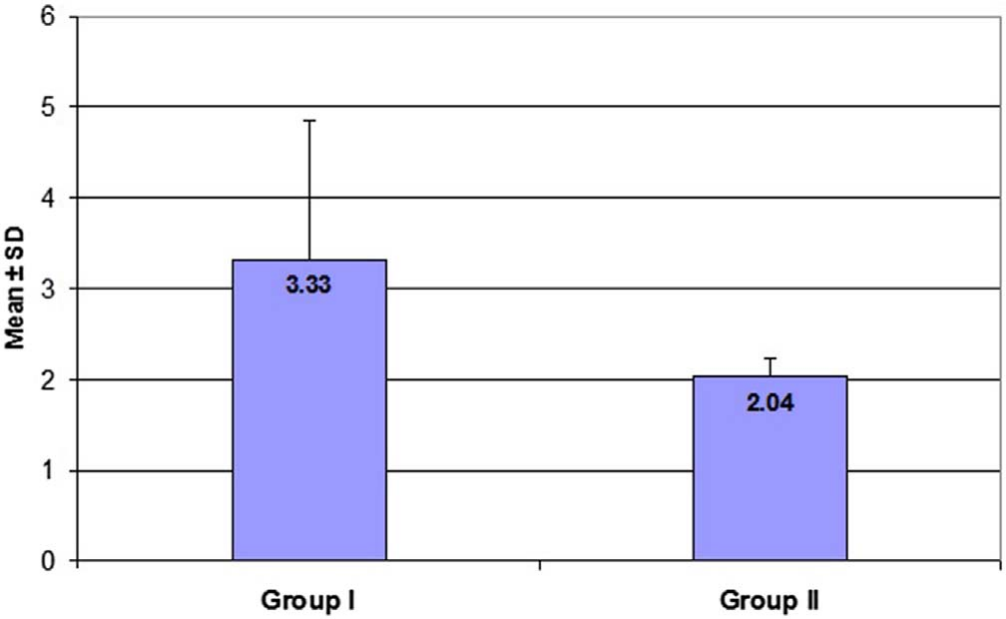
Post-operative hospital stay (complete days). Group I: audit group, Group II: re-audit group.

**Figure 3:**
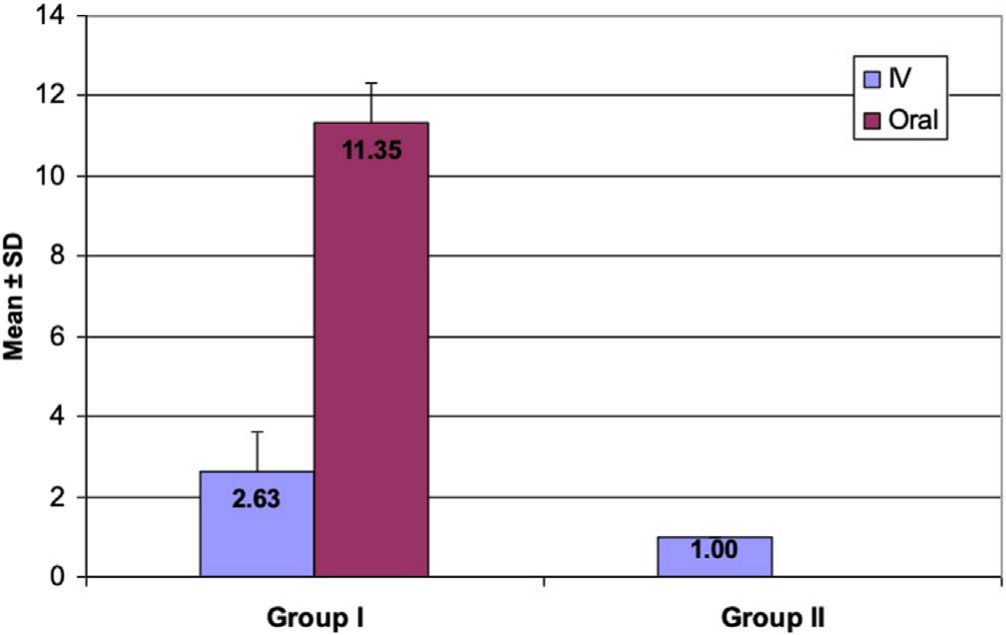
Duration of postoperative antibiotics (complete days). Group I: audit group, Group II: re-audit group.

Following identification of the gaps between the locally used protocols and the international guidelines, the authors performed intensive discussion and agreement with all stakeholders in our department. We could change the local SAP protocol and strictly adhere to using postoperative first-generation cephalosporins prophylactic antibiotics for one day and to discharge all patients out of hospital immediately afterwards (Figure 2). The total hospital stay duration was significantly shorter in group II than in group I (Figure 3).

Out of the 54 patients who underwent clean non-instrumented lumbar spinal surgeries during the audit period (Group I), 39 cases had lumbar discectomy, 8 cases had lumbar decompression, and 7 cases had lumbar discectomy and decompression. SSI was detected during follow-up period, which extended to three months in only one case (1.85%).

Out of the 54 patients who underwent clean non-instrumented lumbar spinal surgeries during the re-audit period (Group II), 38 cases had lumbar discectomy, 9 cases had lumbar decompression, and 7 cases had lumbar discectomy and decompression. No single case of SSI was detected during the follow-up period, which extended to three months.

All patients during the re-audit period were given 1 gm parenteral antibiotic prophylaxis starting 30 min before surgery, using first-generation cephalosporin, which was continued for only 24 h postoperatively and all patients were discharged from the hospital on the second day (Figure 3). Two cases were excluded from the re-audit group because they received antibiotics for other concomitant reasons (upper respiratory tract infection).

There were no intraoperative complications reported in both groups.

## Discussion

SSIs are the most reported type of hospital-acquired infections [12]. Antibiotic stewardship programs are gaining wide acceptance and play important restrictive and persuasive roles to limit overuse of antimicrobials [9, 12, 15]. Published meta-analyses reported no evidence for the additional benefit of prolonged use of antibiotic surgical prophylaxis [9, 16, 17].

This work summarized an audit cycle that convinced orthopedic surgeons in our local setting that obeying the standard guidelines of restricted antibiotic use will not affect the outcomes in clean non-instrumented lumbar spinal surgeries and the ideal practice. This prospective complete audit cycle succeeded to identify the gap, improve local awareness, implement change, and re-audit that change in practice with excellent outcomes.

International guidelines support the use of narrow-spectrum antibiotics for surgical prophylaxis, first-generation cephalosporins are the most widely accepted choice in spinal surgery because it is active against staphylococcal species which are the most common causative pathogens for postoperative infections [2, 18].

A first-generation cephalosporin is relatively nontoxic and inexpensive, and it provides good soft tissue and bone penetration. Also, first-generation cephalosporin has been proved to be more cost-beneficial compared with other wide-spectrum antibiotics such as Ampicillin–sulbactam [19]. The audit results showed that the combination (Amoxicillin–Clavulanic acid) was commonly used in our center for prophylaxis.

The current rapid recovery and fast track trends in lumbar disc surgery enhance discharge to home the day of surgery or on the first postoperative day [20–22]. At our institution, patients undergoing lumbar discectomy were given a single preoperative dose of prophylactic antibiotics directed against gram-positive organisms. Antibiotics were administered approximately 30 min before the surgical starting time. However clinical studies comparing the single preoperative dose with the multiple postoperative doses failed to show any statistical difference [17, 23]. According to the Centre for Disease Control and Prevention (CDC) guidelines, the SAP period is changed to the day of surgery only [4, 24].

Dimick et al. [25] reported that patients should receive the antibiotic within 30 min before skin incision and that antimicrobial prophylaxis (AP) should not be continued longer than 24 h perioperatively, in another study Kim et al. [26] reported that the effectiveness of 48 h antimicrobial treatment was compared with that of 72 h dosage and it was recommended that AP for 48 h is as effective as that for 72 h. Dobzyniak et al. [4] reported that no difference in the rate of infection between patients receiving preoperative antibiotics alone versus those receiving preoperative followed by postoperative antibiotics. However, a recent study [23] concluded that for instrumented spinal surgery, a 72-h antibiotic administration was associated with significantly less incidence of SSI.

There were 103 patients (95.4%) with BMI less than 30 and 5 patients (4.6%) with BMI more than 30 with no SSI. This confirms reports from Rihn et al. [27], Ahmed et al. [28], and Gepstein et al. [29] that obese patients undergoing lumbar discectomy, lumbar decompression, and surgical procedures for lumbar disc herniation achieve similar benefits to non-obese patients.

However, Klemenscics et al. [30] reported significantly increased risk of SSI was associated with BMI higher than 28 kg/m^2^. Bono et al. [31] found that there is a significant increase in complications, specifically infection and surgical complications, in patients with BMI ≥ 35 following lumbar spine surgery, with that rate further increasing with BMI ≥ 40.

In Egypt, SAP is used for prolonged duration and in 2011 a study discussed the inappropriate antibiotic use in 18 hospitals in Egypt and found that the most common indication for antibiotic use, observed in 38.4% of antibiotic prescriptions, was surgical prophylaxis, prolonging the duration of antibiotic prophylaxis and selecting a broad-spectrum agent for prophylaxis were practices commonly used to reduce the risk of SSIs and other healthcare-associated infections in the post-operative period [32]. A definite local protocol for SAP is unfortunately unavailable.

This study highlighted a common hidden problem in a limited resources country. The authors believe that this unjustified and extended use of perioperative antibiotics in spinal surgeries is more common than what has been reported, and involves all kinds of clean surgeries, not only in their institute/country but also in many other countries. This work also should that continuous audit of our practice is an important way to change wrong beliefs and improvement of practice. Furthermore, it calls upon some similar national and international audits to rectify the perioperative use of antibiotics and other areas of malpractice.

In conclusion, a single-day postoperative dose of antibiotics effectively prevents postoperative wound infection following non-instrumented lumbar spinal surgery and is not associated with a higher infection rate. This unnecessary hidden malpractice of long antibiotic use should be abandoned for this indication.

## Disclosure statement

The authors did not receive any funding and have no conflicts related to this study.

## Conflict of interest

All authors declare that they have no conflicts of interest.

## References

[1] Hampson F, Ridgway E (2005) Prophylactic antibiotics in surgery. Surg-Oxford Int Ed 23(8), 290–293.

[2] Mangram AJ, Horan TC, Pearson ML, Silver LC, Jarvis WR, Committee HICPA (1999) Guideline for prevention of surgical site infection, 1999. Infect Cont Hosp Epidemiol 20(4), 247–280. 10.1086/501620 10219875

[3] Anderson DJ, Podgorny K, Berrios-Torres SI, Bratzler DW, Dellinger EP, Greene L, Nyquist AC, Saiman L, Yokoe DS, Maragakis LL, Kaye KS (2014) Strategies to prevent surgical site infections in acute care hospitals: 2014 update. Infect Cont Hosp Epidemiol 35(6), 605–627. 10.1086/676022 PMC4267723 24799638

[4] Dobzyniak MA, Fischgrund JS, Hankins S, Herkowitz HN (2003) Single versus multiple dose antibiotic prophylaxis in lumbar disc surgery. Spine 28(21), E453–E455. 14595175 10.1097/01.BRS.0000090839.61893.BE

[5] Boston KM, Baraniuk S, O’heron S, Murray KO (2009) Risk factors for spinal surgical site infection, Houston, Texas. Infect Cont Hosp Epidemiol 30(9), 884–889. 10.1086/605323 19642902

[6] Scottish IGN (2008) SIGN guideline 58: Safe sedation of children undergoing diagnostic and therapeutic procedures. Paediatr Anaesth 18(1), 11. 18095959 10.1111/j.1460-9592.2007.02405.x

[7] Shaffer WO, Baisden JL, Fernand R, Matz PG (2013) An evidence-based clinical guideline for antibiotic prophylaxis in spine surgery. Spine J 13(10), 1387–1392. 23988461 10.1016/j.spinee.2013.06.030

[8] Mathur P, Trikha V, Farooque K, Sharma V, Jain N, Bhardwaj N, Sharma S, Misra M (2013) Implementation of a short course of prophylactic antibiotic treatment for prevention of postoperative infections in clean orthopaedic surgeries. Ind J Med Res 137(1), 111. PMC3657872 23481059

[9] Davey P, Brown E, Charani E, Fenelon L, Gould IM, Holmes A, Ramsay CR, Wiffen PJ, Wilcox M (2013) Interventions to improve antibiotic prescribing practices for hospital inpatients. Cochrane Database Syst Rev 4, CD003543. 10.1002/14651858.CD003543.pub3 23633313

[10] Fridkin S, Baggs J, Fagan R, Magill S, Pollack LA, Malpiedi P, Slayton R, Khader K, Rubin MA, Jones M, Samore MH, Dumyati G, Dodds-Ashley E, Meek J, Yousey-Hindes K, Jernigan J, Shehab N, Herrera R, McDonald CL, Schneider A, Srinivasan A, Centers for Disease C, Prevention (2014) Vital signs: Improving antibiotic use among hospitalized patients. MMWR Morb Mortal Wkly Rep 63(9), 194–200. 24598596 PMC4584728

[11] Michael CA, Dominey-Howes D, Labbate M (2014) The antimicrobial resistance crisis: Causes, consequences, and management. Front Public Health 2, 145. 25279369 10.3389/fpubh.2014.00145 PMC4165128

[12] Segala FV, Murri R, Taddei E, Giovannenze F, Del Vecchio P, Birocchi E, Taccari F, Cauda R, Fantoni M (2020) Antibiotic appropriateness and adherence to local guidelines in perioperative prophylaxis: Results from an antimicrobial stewardship intervention. Antimicrob Resist Infect Control 9(1), 164. 33106190 10.1186/s13756-020-00814-6 PMC7586646

[13] Horan TC, Gaynes RP, Martone WJ, Jarvis WR, Emori TG (1992) CDC definitions of nosocomial surgical site infections, 1992: A modification of CDC definitions of surgical wound infections. Infect Control Hosp Epidemiol 13(10), 606–608. 1334988

[14] Kailash KK, Vijayraghavan P (2013) Prospective randomized study for antibiotic prophylaxis in spine surgery: Choice of drug, dosage, and timing. Asian Spine J 7(3), 196–203. 24066215 10.4184/asj.2013.7.3.196 PMC3779771

[15] Barlam TF, Cosgrove SE, Abbo LM, MacDougall C, Schuetz AN, Septimus EJ, Srinivasan A, Dellit TH, Falck-Ytter YT, Fishman NO, Hamilton CW, Jenkins TC, Lipsett PA, Malani PN, May LS, Moran GJ, Neuhauser MM, Newland JG, Ohl CA, Samore MH, Seo SK, Trivedi KK (2016) Implementing an Antibiotic Stewardship Program: Guidelines by the Infectious Diseases Society of America and the Society for Healthcare Epidemiology of America. Clin Infect Dis 62(10), e51–e77. 27080992 10.1093/cid/ciw118 PMC5006285

[16] Kim B, Moon SH, Moon ES, Kim HS, Park JO, Cho IJ, Lee HM (2010) Antibiotic microbial prophylaxis for spinal surgery: Comparison between 48 and 72-hour AMP protocols. Asian Spine J 4(2), 71–76. 21165308 10.4184/asj.2010.4.2.71 PMC2996630

[17] Yao R, Tan T, Tee JW, Street J (2018) Prophylaxis of surgical site infection in adult spine surgery: A systematic review. J Clin Neurosci 52, 5–25. 29609860 10.1016/j.jocn.2018.03.023

[18] Pharmacists ASoH-S (1999) ASHP therapeutic guidelines on antimicrobial prophylaxis in surgery. American Society of Health-System Pharmacists. Am J Health-Syst Pharm 56(18), 1839–1888. 10511234 10.1093/ajhp/56.18.1839

[19] Pawar AY, Biswas SK (2016) Postoperative spine infections. Asian Spine J 10(1), 176. 26949475 10.4184/asj.2016.10.1.176 PMC4764532

[20] Dietz N, Sharma M, Adams S, Alhourani A, Ugiliweneza B, Wang D, Nuno M, Drazin D, Boakye M (2019) Enhanced recovery after surgery (ERAS) for spine surgery: A systematic review. World Neurosurg 130, 415–426. 31276851 10.1016/j.wneu.2019.06.181

[21] Fleege C, Arabmotlagh M, Almajali A, Rauschmann M (2014) Pre- and postoperative fast-track treatment concepts in spinal surgery: Patient information and patient cooperation. Orthopade 43(12, 1062–1064, 1066–1069. 25387654 10.1007/s00132-014-3040-5

[22] Fleege C, Rauschmann MA (2014) Treatment strategies to shorten convalescence after spinal surgery : From treatment begin to recovery. Orthopade 43(12), 1041–1042. 25403692 10.1007/s00132-014-3046-z

[23] Maciejczak A, Wolan-Nieroda A, Walaszek M, Kolpa M, Wolak Z (2019) Antibiotic prophylaxis in spine surgery: A comparison of single-dose and 72-hour protocols. J Hosp Infect 103(3), 303–310. 31051190 10.1016/j.jhin.2019.04.017

[24] Rubinstein E, Findler G, Amit P, Shaked I (1994) Perioperative prophylactic cephazolin in spinal surgery. A double-blind placebo-controlled trial. J Bone Joint Surg Br 76(1), 99–102. 8300691

[25] Dimick JB, Lipsett PA, Kostuik JP (2000) Spine update: Antimicrobial prophylaxis in spine surgery: Basic principles and recent advances. Spine 25(19), 2544–2548. 11013510 10.1097/00007632-200010010-00020

[26] Kim B, Moon S-H, Moon E-S, Kim H-S, Park J-O, Cho I-J, Lee H-M (2010) Antibiotic microbial prophylaxis for spinal surgery: Comparison between 48 and 72-hour AMP protocols. Asian Spine J 4(2), 71–76. 21165308 10.4184/asj.2010.4.2.71 PMC2996630

[27] Rihn JA, Kurd M, Hilibrand AS, Lurie J, Zhao W, Albert T, Weinstein J (2013) The influence of obesity on the outcome of treatment of lumbar disc herniation: Analysis of the Spine Patient Outcomes Research Trial (SPORT). J Bone Joint Surg Am 95(1), 1–8. 23192403 10.2106/JBJS.K.01558 PMC3528022

[28] Ahmed A, Venkatesan M, Newey M (2017) Comparison of outcome between obese and non-obese patients after primary lumbar discectomy. Int J Orthop 4(2), 714–718.

[29] Gepstein R, Shabat S, Arinzon Z, Berner Y, Catz A, Folman Y (2004) Does obesity affect the results of lumbar decompressive spinal surgery in the elderly? Clin Orthop Relat Res 426, 138–144. 10.1097/01.blo.0000141901.23322.98 15346065

[30] Klemencsics I, Lazary A, Szoverfi Z, Bozsodi A, Eltes P, Varga PP (2016) Risk factors for surgical site infection in elective routine degenerative lumbar surgeries. Spine J 16(11), 1377–1383. 27520077 10.1016/j.spinee.2016.08.018

[31] Bono OJ, Poorman GW, Foster N, Jalai CM, Horn SR, Oren J, Soroceanu A, Ramachandran S, Purvis TE, Jain D, Vira S, Diebo BG, Line B, Sciubba DM, Protopsaltis TS, Buckland AJ, Errico TJ, Lafage V, Bess S, Passias PG (2018) Body mass index predicts risk of complications in lumbar spine surgery based on surgical invasiveness. Spine J: Off J North Am Spine Soc 18(7), 1204–1210. 10.1016/j.spinee.2017.11.015 29155339

[32] Talaat M, Saied T, Kandeel A, El-Ata G, El-Kholy A, Hafez S, Osman A, Razik M, Ismail G, El-Masry S (2014) A point prevalence survey of antibiotic use in 18 hospitals in Egypt. Antibiotics 3(3), 450–460. 27025755 10.3390/antibiotics3030450 PMC4790372

